# Notes and News

**Published:** 1905-04-01

**Authors:** 


### Notes and News.

The proceeds of the Ascot Ball, on June 15th, are to be devoted to the Victoria Hospital for Children, Chelsea.

The Secretary of the Royal Waterloo Hospital for Women and Children makes an earnest appeal for the gift of letters to Convalescent Homes for the use of the patients.

In Belfast the general importance of a pure milk supply is focussing public attention, and the Health Committee of that city have pledged themselves to move Parliament, if necessary, to render the regulations framed for dairies of a more stringent nature.

The second assembly of the French Congress of Climatology and Urban Hygiene will be held in Arcachon and the neighbourhood, from April 24th to 29th, when the climate and natural conditions will be studied, excursions being made to all places of interest. Members will be entitled to a reduction in fares, and all particulars may be obtained from Dr. Dechamp, Villa Tibur, Arcachon.

We hear from Miss M. Horne, who has invented a hammock for invalid travellers adaptable to railway carriages and other conveyances, that two saloon carriages on the Great Northern Railway have been fitted with the appliance. The adjustment is very simple, and the whole cost of installation very small, and as a comfortable and inexpensive mode of moving invalids to health resorts it certainly should be adopted on all the railways.

The present law in France in relation to milk from tuberculous cows is not satisfactory from a hygienic point of view. Milk may be sold in France if the tuberculous lesions in the cow are not very marked, and provided the teats are not affected. The value of distinctions drawn is certainly open to question. Attention has been drawn to the matter recently, and the experiments which have been made are disquieting in the results.

The late Miss Ellen Ware, of Tunbridge Wells, has bequeathed £500 each to the General Hospital, Tunbridge Wells, Guy's Hospital, St. Thomas's Hospital, the Hospital for Women, Soho Square, and the Hospital for Incurables, Putney Heath.

The Windsor and Egham Joint Hospital Board have before them a proposal to erect an infectious diseases hospital. The question is surrounded with *pros* and *cons*. There is an offer of a site on Crown lands, but to that is attached conditions which affect the price. Some of the Board are in favour of cheap bungalow wards, but as the hospital will be within view of Windsor Castle the doubt is expressed whether such would be permitted. In the discussion as to the cost per bed which should be incurred in the construction the opinions varied to a remarkable degree. One gentleman quoted £40, and another stood by a minimum of £500. So many isolation hospitals have been established lately that a small committee of inquiry should have no difficulty in finding an example that will serve to guide the Board to secure efficiency at a reasonable cost.

At the annual meeting of the Governors of the Metropolitan Convalescent Institution held recently Mr. M. O. FitzGerald presided, and it was reported that the first portion of a new home for men, at Little Common, Bexhill, erected from the design of Mr. Rowland Plumbe, at an estimated cost, including the freehold site, of £22,423, of which £16,666 was provided by the bequest of the late Mr. Henry Vaughan, would be ready for occupation in May. The present building will accommodate 71 patients, and when completed, at an extra cost of £6,500, it will provide for 118 patients. The additional accommodation is urgently needed, and an earnest appeal is made for the £10,000 required for the completion of the home and the liquidation of the balance due to the contractors for the part already built, and also for an increased income of £900 for the maintenance of the homes.

The statements made by Dr. Phillips at a recent quarterly meeting of the Hanley, Stoke, Fenton and Longton Joint Hospital Board contain sufficient reasons to decide the Board that an isolation hospital should be provided forthwith. The plea which is raised in so many places that cases of infectious disease are so few that a hospital would be empty most of the time cannot be brought forward on behalf of the locality round Hanley. Dr. Phillips said that enteric fever had assumed an epidemic condition in the Potteries for some years; and the North Staffordshire Infirmary had set aside 16 beds for its treatment, but that such a number could not be considered in so extensive a district. Dr. Phillips pointed out moreover that the arrangement was not in accordance with modern ideas. He suggested the provision of a pavilion of 20 beds, and gave details of the approximate cost of establishment and maintenance. Postponement of the matter under consideration was decided upon with the avidity very frequently exhibited when such a course is proposed in connection with similar deliberations, and for 12 months more Hanley has pledged itself not to think of the disagreeable topic.

